# A diagnostic pitfall in psoriasiform lesions: From psoriasis to dermatomyositis

**DOI:** 10.1016/j.jdcr.2026.08.005

**Published:** 2026-08-17

**Authors:** Paulus Mario Christopher, Ferra Olivia Mawu, Meilany Feronika Durry, Aryani Adji

**Affiliations:** a Department of Dermatology, Venereology, and Aesthetics, Faculty of Medicine, Sam Ratulangi University/ Prof. Dr. R. D. Kandou General Hospital, Manado, North Sulawesi, Indonesia; b Department of Anatomical Pathology, Faculty of Medicine, Sam Ratulangi University/ Prof. Dr. R. D. Kandou General Hospital, Manado, North Sulawesi, Indonesia

**Keywords:** autoimmune muscle disease, dermatomyositis, inflammatory disease, psoriasiform, small ubiquitin-like modifier activating enzyme (SAE)

## Case description

A 64-year-old retired policeman presented with a 6-month history of intensely pruritic, painful, photosensitive erythematous plaques that gradually progressed to near-generalized psoriasiform lesions, initially misdiagnosed and unsuccessfully treated as psoriasis vulgaris.

On reevaluation, he had generalized violaceous-erythematous macules and plaques with white dry scale, erosions, excoriations, and xerosis, accompanied by classic dermatomyositis stigmata, including heliotrope rash, Gottron papules and Gottron sign, V-neck sign, shawl sign, holster sign, poikiloderma, mechanic’s hands (Fig 1, *A*-*J*), and an ovoid palatal patch. Calcinosis cutis was observed on both forearms. Dermoscopy and trichoscopy were supportive of dermatomyositis (Fig 2, *A*-*D*). Neurological workup revealed no subjective or objective muscle weakness.

**Fig 1:**
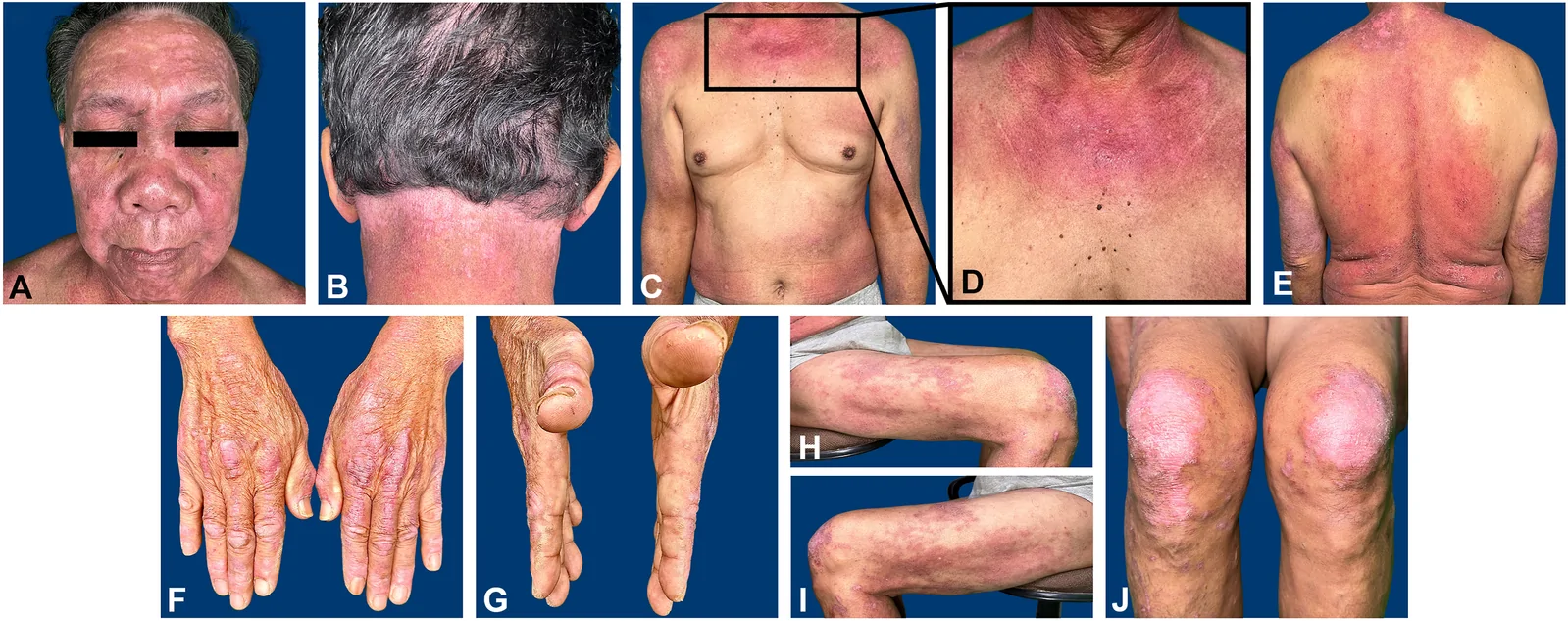
Clinical images of the face revealing **(A)** heliotrope rash and erythematous-violaceous macules on the nasolabial folds, **(B)** posterior hair line, **(C** and **D)** V-neck sign, **(E)** shawl sign and poikiloderma, **(F)** Gottron papules, **(G)** Mechanic’s hands, **(H** and **I)** holster sign, and **(J)** Gottron sign.

**Fig 2:**
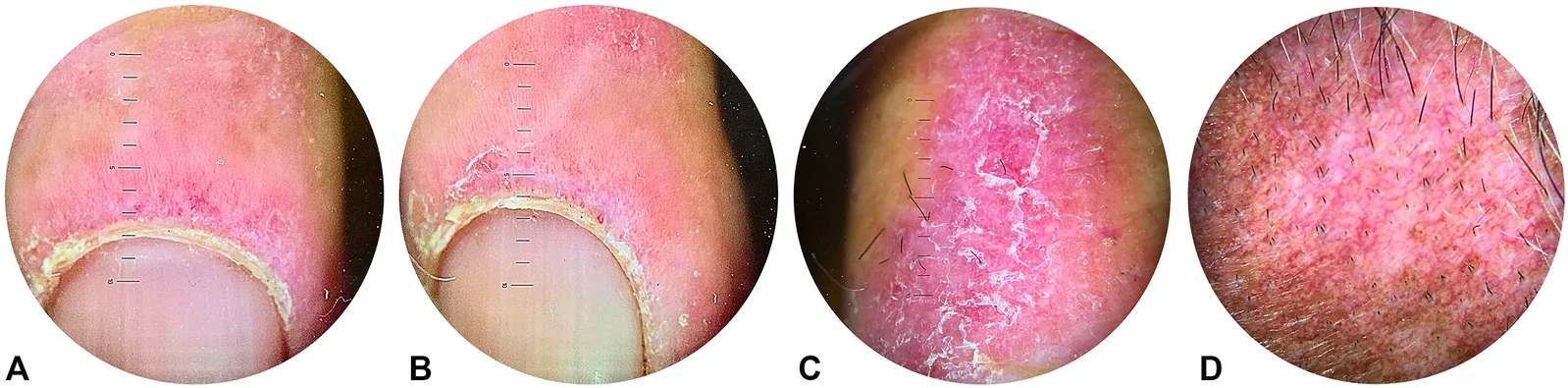
**A** and **B,** Proximal nailfold dermoscopy revealed capillary telangiectasia, avascular area, and giant capillaries, **(C)** dermoscopy of Gottron papule revealed irregularly distributed dots and linear vessels surrounded by structureless white areas, and **(D)** scalp trichoscopy showed yellow dots, an interfollicular honeycomb pigment pattern, and thick linear, branching, and tortuous capillaries.

Laboratory studies demonstrated an elevated erythrocyte sedimentation rate (120 [2-30] mm/h), creatine kinase (239 [26-190] U/L), and lactate dehydrogenase (304 [91-232] U/L), with a high-titer speckled antinuclear antibody. Histopathological examination revealed interface dermatitis (Fig 3, *A*-*D*). Chest radiography, electrocardiography, and malignancy screening (prostate-specific antigen and carcinoembryonic antigen) were unremarkable.

**Fig 3:**
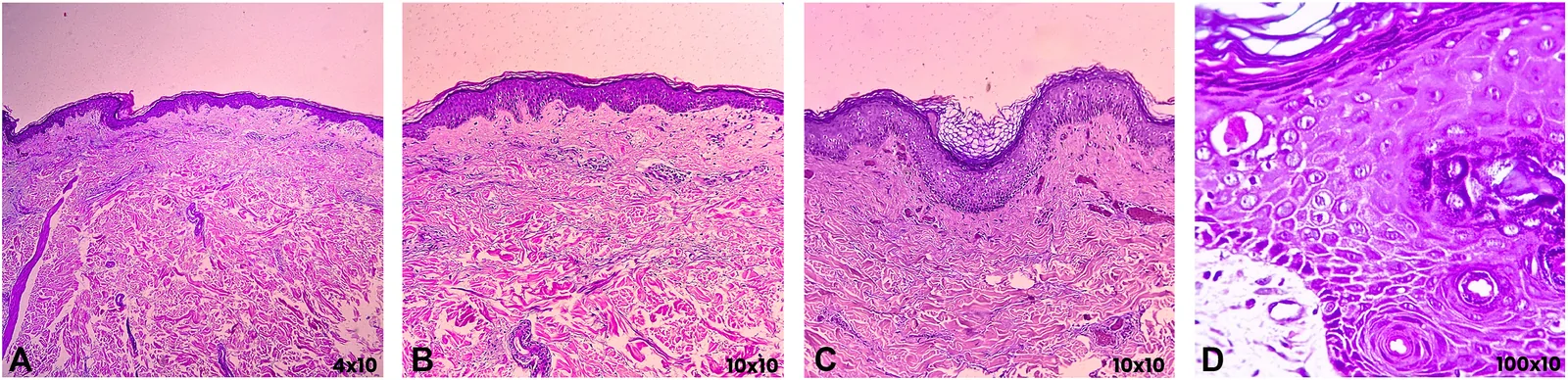
**A-D,** Histopathological findings show an interface dermatitis, atrophic epidermis with hyperkeratosis, parakeratosis, keratotic plaques, cytoid bodies, vacuolar degeneration of basal keratinocytes, focal pigment incontinence, and superficial dermal mucin deposition **(**Hematoxylin-eosin stain).

Combination therapy with tapering systemic corticosteroids, weekly methotrexate, and topical corticosteroids resulted in gradual improvement in erythema, scales, pruritus, and laboratory abnormalities over 6 months.

**Question: Which myositis-specific autoantibody is most characteristic of this patient’s skin-predominant, intensely pruritic erythema with minimal clinical myositis?**

**A.** Anti-Mi-2
**B.** Anti-MDA5
**C.** Anti-TIF1-γ
**D.** Anti-SAE
**E.** Anti-NXP2

## Answer and discussion

The correct answer is **anti-SAE**. Anti-small ubiquitin-like modifier activating enzyme (anti-SAE) is a rare myositis-specific autoantibody that defines a distinct dermatomyositis subset with striking, often intensely pruritic, diffuse cutaneous erythema and psoriasiform or erythrodermic eruptions that may precede or occur in the absence of significant clinical myositis.^1^^,^^2^ The patient’s photosensitive violaceous erythema, heliotrope rash, Gottron papules and Gottron sign, V-neck and shawl signs, holster sign, poikiloderma, mechanic’s hands, and nailfold capillary changes are pathognomonic for dermatomyositis rather than psoriasis.^1^ Interface dermatitis with vacuolar alteration and dermal mucin further supports an autoimmune connective tissue process instead of psoriasiform hyperplasia or cutaneous T-cell lymphoma. Although calcinosis cutis and an ovoid palatal patch may be seen in other myositis-specific autoantibody subtypes such as anti-NXP2 and anti-TIF1-γ disease, respectively,^1^ this patient’s strongly positive anti-SAE antibodies with negative testing for other myositis-specific autoantibodies confirms classification as anti-SAE dermatomyositis.

Anti-SAE dermatomyositis is a rare myositis-specific autoantibody subtype, with reported prevalence generally below 10% across dermatomyositis cohorts.^3^ Anti-SAE positivity has been linked to mild muscle involvement, possible interstitial lung disease, and an increased risk of adenocarcinoma, warranting ongoing malignancy surveillance even when baseline screening is unrevealing.^1^^,^^2^ Mechanistically, SAE participates in SUMOylation, a post-translational modification regulating type I interferon signaling, NF-κB activation, and T-cell responses, offering a plausible explanation for the skin-predominant inflammatory phenotype in anti-SAE dermatomyositis.^4^^,^^5^ There are no treatment guidelines specific to this subset; management generally follows conventional dermatomyositis protocols, combining systemic corticosteroids with steroid-sparing immunosuppressants, such as methotrexate or mycophenolate mofetil.^1^

This case highlights that in older patients with treatment-refractory psoriasiform lesions, careful attention to photodistribution, dermatomyositis stigmata, supported by dermoscopy, histopathology, and early myositis-specific autoantibody testing, is essential to prevent misdiagnosis and facilitate timely immunosuppression and malignancy surveillance.

### Declaration of generative AI and AI-assisted technologies in the writing process

None.

## Conflicts of interest

None disclosed.
